# Bortezomib Inhibits Multiple Myeloma Cells by Transactivating ATF3 to Trigger miR-135a-5p- Dependent Apoptosis

**DOI:** 10.3389/fonc.2021.720261

**Published:** 2021-09-22

**Authors:** Xiaolan Lai, Chuanqian Huang, Xuekun Nie, Qi Chen, Yirong Tang, Xianguo Fu, Ying Lin, Chengjun Nie, Xinyu Xu, Xiukang Wang, Renli Chen, Zichun Chen

**Affiliations:** ^1^Department of Hematology and Rheumatism, Ningde Municipal Hospital Affiliated to Ningde Normal University, Ningde, China; ^2^Department of Medical Oncology and Radiotherapy, Ningde Municipal Hospital Affiliated to Ningde Normal University, Ningde, China; ^3^Department of Pharmacy, Ningde Municipal Hospital, Affiliated to Ningde Normal University, Ningde, China; ^4^Central Laboratory, Ningde Municipal Hospital Affiliated to Ningde Normal University, Ningde, China

**Keywords:** multiple myeloma, bortezomib, miR-135a-5p, ATF3, apoptosis

## Abstract

Multiple myeloma (MM) is a malignant cancer with an increasing in incidence that can be alleviated through bortezomib (BTZ) treatment. Activating transcription factor 3 (ATF3) plays a major role in cancer development. Moreover, microRNAs (miRNAs) regulate carcinogenic pathways, apoptosis, and programmed necrotic cell death. However, the detailed mechanism by which ATF3 modulates BTZ drug sensitivity/resistance remains elusive. In the current study, expression of ATF3 was significantly increased under BTZ treatment in a dose-dependent manner in MM cell lines. In addition, ATF3 could regulate cell apoptosis under BTZ treatment. The effect of ATF3 was negatively regulated by its binding miRNA, miR-135a-5p. When either ATF3 was silenced or miR-135a-5p mimics were added to MM cells, they partially lost sensitivity to BTZ treatment. This was accompanied by low levels of Noxa, CHOP, and DR5, and a decrease in mitochondrial membrane potential. These results revealed the combinatorial regulatory patterns of ATF3 and miR-135a-5p in the regulatory protein interactome, which indicated a clinical significance of the miR-135a-5p-ATF3 protein interaction network in BTZ therapy. This study provides potential evidence for further investigation into BTZ resistance.

## Introduction

As a primary bone cancer, multiple myeloma (MM) commonly occurs in the elderly population (aged ≥70 years), which has an increasing incidence and can cause hematologic malignancy (1, 2). Currently, the therapeutic options for MM patients are mainly based on age, tumor stage, and comorbidities (3). A frequently used chemotherapeutic drug in MM is bortezomib (BTZ), it acts as a proteasome inhibitor and interferes with the degradation of misfolded proteins (4, 5). BTZ suppresses ubiquitin-dependent protein degradation and preferentially kills various tumor cells (6). BTZ also sensitizes cells to oxidative damage by the iron status to enhance proteasome inhibition (7, 8). Despite the unsurpassed anti-tumor activity of BTZ for MM (9), drug resistance and a lack of sensitivity to BTZ have restricted the clinical application and patient benefits (10). Thus, elucidation of the mechanism by which BTZ drug sensitivity/resistance occurs in MM would facilitate development of treatments for this disease.

Activating transcription factor (ATF) has been reported to be disrupted in BTZ-treated MM cells (5). After silencing ATF3 or ATF4 expression, MM cells partially lose sensitivity to BTZ treatment and have decreased levels of Noxa, C/EBP-homologous protein (CHOP), and Death receptor 5 (DR5) (11). Additionally, bioinformatics analysis of MM microarray data (GSE13591) has shown that ATF3 is deregulated in this disease (12). Overexpression of ATF3, ATF4, and ATF5 induced by BTZ treatment is associated with sensitivity to BTZ-induced apoptosis in B-lymphoma cells (13). Low levels of XBP1, ATF3, and ATF4 are observed in poor responders to BTZ treatment (14). Upregulation of chemokine (C-C motif) ligand 2 (CCL2) resulting from an enhanced interaction between c-Jun and ATF3 in the brain contributes to BTZ-induced mechanical allodynia (4). However, the detailed mechanism by which BTZ regulates ATF3 remains elusive.

Aberrant expression of microRNAs (miRNAs) has been reported to be a molecular pathogenesis of MM. For example, miR-155 and miR-33b expression may elicit anti-MM activity, likely *via* proteasome inhibition (15, 16). A study shown that miR-22 negatively regulates the anti-apoptotic protein phosphoprotein enriched in diabetes (PED) and tumor necrosis factor-related apoptosis-inducing ligand-induced cell death in non-small cell lung cancer cells (17). The potential of miRNAs to participate in BTZ effectiveness and resistance was provided insights into promising therapeutic options for patients with MM (18). Combined overexpression of miR-520g and miR-520h exerts an inhibitive effect on BTZ-resistant MM tumor growth *in vivo (* 19). MiR-200c is capable of enhancing BTZ-induced cancer cell death through the pro-apoptotic Bcl-2 family member Noxa (20). Furthermore, miR-29b has been reported to be a potentiator that synergistically promotes the anti-myeloma effect of BTZ (10).

Interestingly, expression of ATF3 is closely associated with miRNAs such as miR-488 (21). miR-342-3p directly targets ATF3 to modulate transcription of pro-osteogenic differentiation-associated genes (22). MiR-494 affects ATFs through binding to its 3′UTR (23). However, in MM, miRNAs-ATF3 regulatory protein interaction network in BTZ therapy is unclear. In this study, we investigated the detailed mechanisms by which BTZ modulates MM cells and demonstrate an interaction between ATF3 and miRNA in BTZ actions.

## Materials and Method

### Cell Culture

Human MM cell linea RPMI-8226 and U-266 were obtained from Xiamen Immocell Biotechnology Co. Ltd. (China). Cells were cultured in RPMI-1640 medium with 10% FBS (fetal bovine serum), 100 U/mL penicillin and streptomycin each, and 2 mM L-glutamine at 37°C in a 5% CO_2_ incubator.

### Cell Proliferation Assay and IC_50_ Measurement

A total of 3 × 10^4^ cells/well were seeded in 96-well plates. After treatment with various concentrations of BTZ (S1013, Selleck) at 37°C for 24 h, cell proliferation was measured using an MTT cell proliferation assay kit (Beyotime, Shanghai). Absorbance was measured at 490 nm using a SpectraMax Absorbance Reader (Molecular Devices, San Francisco, CA, USA). The experiment was repeated three times.

### Reverse Transcription-Quantitative PCR Assay

RNA extracted from the cells was subjected to reverse transcription (RT) using Superscript III Reverse Transcriptase (Invitrogen, Thermo Fisher Scientific, Inc.) at 50°C for 45 min. qPCR was conducted using a Bio-Rad CFX96 system and ChamQ SYBR^®^ qPCR Master Mix kit (Vazyme Biotech Co., Ltd.) to determine the mRNA or miRNA expression levels of the genes of interest. Quantitation was performed as described previously (24). The thermocycling conditions were 98°C for 2 min, followed by 30 cycles of 98°C for 10 s, 60°C for 25 s, and 72°C for 25 s. Each reaction was performed in triplicate. Expression levels were normalized to those of U6 or 18S ribosomal RNA. The primers used for qPCR are listed in **Supplemental Table 1**.

### Western Blotting

Cells were lysed in ice-cold radioimmunoprecipitation assay buffer (Sangon Biotech Co., Ltd.). The total protein was quantitated using a BCA Protein Assay kit (Abcam). Lysates (20 mg/sample) were resolved by 8%–12% denaturing SDS-PAGE and the proteins were transferred to a PVDF membrane (Roche Diagnostics GmbH). Tris-HCl buffer with 5% bovine serum albumin (BSA; Beijing Solarbio Science & Technology Co., Ltd.) was used to block the membranes at 28°C for 2 h. The membrane was probed with primary antibodies in Tris-HCl buffer with 5% BSA at 4°C overnight. Subsequently, the membranes were washed three times for 5 min each wash with Tris-HCl buffer that contained 0.1% Tween-20, followed by incubation with the corresponding secondary antibodies in Tris-HCl buffer at 28°C for 1 h. Finally, the membranes were washed three times for 5 min each wash, developed, and visualized using an enhanced chemiluminescence detection system (Thermo Fisher Scientific, Inc.). The antibodies used in this study were anti-ATF3 (1:500), anti-GAPDH (1:5,000; cat. no. YM3029; ImmunoWay Biotechnology), HRP-conjugated goat anti-rabbit IgG (1:1,000; cat. no. 7074; Cell Signaling Technology, Inc.), and HRP-conjugated rabbit anti-mouse IgG (1:1,000; cat. no. 7076; Cell Signaling Technology, Inc.). Detailed information on the antibodies is shown in **Supplemental Table 2**. Western blot densitometry was performed using ImageJ software.

### Plasmid Construction and Transfection

For ATF3 knockdown, human ATF3-targeting shRNA sequences were cloned into a plko.1 vector. primers for construction of plko.1-shATF3 plasmid were synthesized from Ribobio (Guangzhou, Guangdong, China) and the sequences were shown in **Supplemental Table 3**. A control shRNA unrelated to the human sequences was used as a negative control.

Genomic DNA of the cells was isolated using a FastPure Cell DNA Isolation Mini kit (cat. no. DC102; Vazyme Biotech Co., Ltd.). The 3′UTR of the human ATF3 gene was amplified by PCR using the genomic DNA with a 2X Phanta Master Mix kit (cat. no. P511-01; Vazyme Biotech Co., Ltd.). The PCR conditions were 98°C for 2 min, followed by 30 cycles of 98°C for 15 s, 60°C for 15 s, and 72°C for 60 s. ATF3, CHOP, DR5, and Noxa 3′UTR fragments were cloned downstream of the firefly luciferase reporter gene in the pmirGLO vector (Promega), to generate pmirGLO-ATF3, -CHOP, -DR5, and Noxa 3′UTR WT plasmids. Mutant ATF3, CHOP, DR5, and Noxa 3′UTRs were generated using a QuikChange II Site-Directed Mutagenesis kit (Agilent Technologies, Inc.) and cloned into the pmirGLO vector to generate the pmirGLO-3′UTR mutant plasmid. The primers for gene cloning are shown in **Supplemental Table 3**.

### Cell Treatments

MM cells were seeded in 6-well plates at a density of 2 × 10^6^/well. Four micrograms of plasmids (shcrtl or shATF3) or 200 pmol mimics (mimic NC or miR-135a-5p mimic) were transfected into cells using Lipofectamine^®^ 3000 (Invitrogen; Thermo Fisher Scientific, Inc.). Six hours after transfection, RPMI-8226 and U-266 cells were treated with 10 or 20 nM BTZ at 37°C for 24 h. After treatment, the cells were harvested and used for reactive oxygen species (ROS) measurement, mitochondrial membrane potential (MMP) detection, and apoptosis assays.

### ROS Detection

The ROS level in cells was measured using a Cellular ROS Assay Kit (ab113851, Abcam) in accordance with the manufacturer’s instructions.

### MMP Detection

A JC-1 staining assay kit (C2006, Beyotime) was used to detect alterations in the mitochondrial membrane potential. Cells were resuspended in 500 μL RPMI-1640 medium and then mixed with 500 μL JC-1 working solution. After incubation for 20 min at 37°C while protect from light, the cells were washed twice with JC-1 staining buffer, and resuspended in 300 μL JC-1 staining buffer, and then analyzed by a NovoCyte FACS (Cat: 1300, ACEA, San Diego, CA, USA).

### Apoptotic Assay

Cells were stained with Annexin V-fluorescein isothiocyanate and 30 mg/mL PI (Cat: A211-02, Vazyme, Nanjing, China) and were then analyzed by FACS and FlowJo software.

### Bioinformatic Analysis

TargetScan (http://www.targetscan.org/vert_72/), miRPathDB (https://mpd.bioinf.uni-sb.de/), DIANA Tools (http://diana.imis.athenainnovation.gr/DianaTools/), and mirDIP (http://ophid.utoronto.ca/mirDIP/index.jsp) databases were used to screen ATF3 targets and binding. Statistical differences in survival rates were analyzed using the log-rank test.

### Actinomycin D Treatment

Act D was purchased from Selleck (S9624, Shanghai, China). Cells were treated with Act D at a concentration in accordance with the IC_50_.

### Dual Luciferase Reporter Assay

MiR-135a-5p mimics and negative control mimics (mimics ctrl) were obtained from Guangzhou RiboBio Co., Ltd. The sequences were miR-135a-5p mimics 5′-UAUGGCUUUUUAUUCCUAUGUGA-3′ and mimics ctrl 5′-UUCUCCGAACGUGUCACGUTT-3′. Cells were seeded in a six-well plate (1×10^6^ cells/well) and transfected with mimics ctrl or miR-135a-5p mimics (200 pmol/well), along with the pmirGLO-WT ATF3 3UTR (4 μg/well) or pmirGLO-Mutant ATF3 3′UTR plasmid (4 μg/well) using Lipofectamine^®^ 3000 (Invitrogen; Thermo Fisher Scientific, Inc.) and incubated at 37°C. The cells were lysed at 50 h post-transfection and luciferase activity was measured using the Dual-Glo Luciferase Assay System (Promega) in accordance with the manufacturer’s instructions. Firefly luciferase activity was normalized to Renilla luciferase activity. Each treatment was carried out in triplicate.

### Statistical Analysis

All statistical analyses were conducted using SPSS version 22.0 (IBM) and GraphPad Prism version 8.0.2 (GraphPad Software, Inc.). Data are presented as the mean ± SD. Differences between two groups were analyzed using the Student’s t-test and one-way ANOVA followed by Tukey’s post-hoc test was used for multiple comparisons among three or more experimental groups. P < 0.05 was considered to indicate a significant difference.

## Results

### BTZ Inhibits Cell Proliferation by Up-regulating ATF3 in a Dose-Dependent Manner

**Figure 1:**
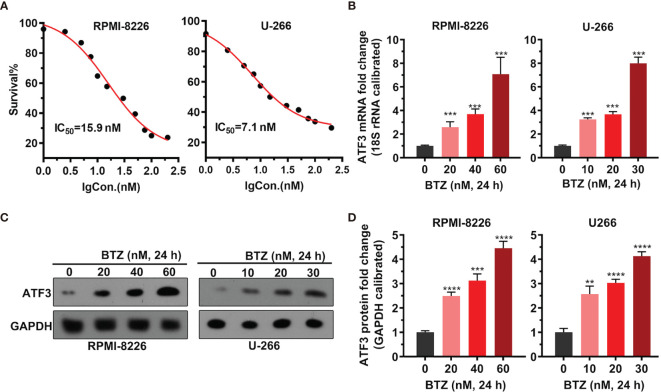
Effects of bortezomib (BTZ) on multiple myeloma (MM) cell proliferation and ATF3 expression. **(A)** MM cell proliferation was assessed by MTT assays. BTZ was used to treat RPMI-8226 and U-266 cells at various doses. **(B)** The ATF3 mRNA level was measured by RT-qPCR in RPMI-8226 and U-266 cells treated with various doses of BTZ. One-way ANOVA was used for statistical analysis. **(C)** The ATF3 protein level was measured by western blotting in RPMI-8226 and U-266 cells treated with various doses of BTZ. **(D)** Quantification of ATF3 band intensity, with values normalized to GAPDH. Data were analyzed by one-way ANOVA, followed by Tukey’s post-hoc test. **P < 0.01, ***P < 0.001, ****P < 0.0001 compared to 0 h.

To examine the dosage effects of BTZ on MM cell proliferation, the proliferation of MM cell lines RPMI-8226 and U-266 under various BTZ treatments was determined by MTT assays. There was a BTZ dose-dependent toxic effect by killing MM cells with an IC_50_ = 15.9 nM in RPMI-8226 cells and an IC_50_ = 7.1 nM in U-266 cells (**Figure 1A**). Additionally, the level of ATF3 mRNA was significantly increased under BTZ treatment in a dose-dependent manner in both MM cell lines (**Figure 1B**). Correspondingly, the protein level of ATF3 was also increased by BTZ (**Figures 1C, D**). These results indicated that BTZ inhibited cell proliferation by upregulating ATF3.

### BTZ Modulates Cell Apoptosis *via* ATF3

**Figure 2:**
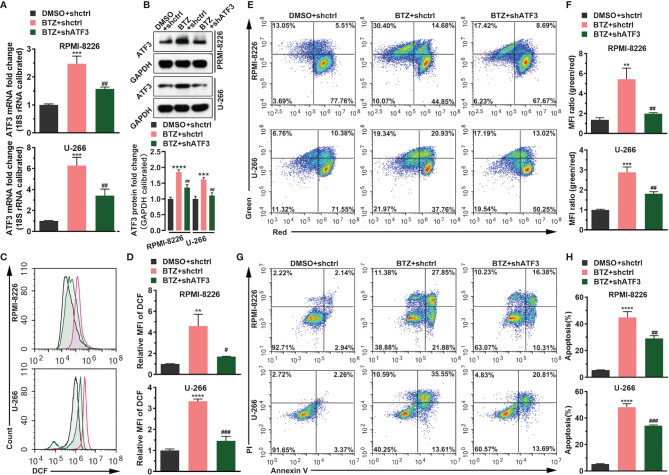
BTZ modulates cell apoptosis *via* ATF3. Apoptosis was analyzed in three groups: **(A)** DMSO + shRNA ctrl; **(B)** bortezomib (BTZ) + shRNA ctrl; **(C)** BTZ + shATF3. **(A)** The ATF3 mRNA level was analyzed by RT-qPCR in RPMI-8226 and U-266 cells of the three groups. **(B)** The ATF3 protein level was analyzed in the three groups. Quantification of ATF3 band intensity, with values normalized to GAPDH. **(C)** The reactive oxygen species (ROS) level was measured by the Cellular ROS Assay Kit in the three groups. 2′,7′-Dichlorofluorescein (DCF) was detected by FACS. **(D)** Quantitation of the ROS level indicated by DCF. **(E)** The mitochondrial membrane potential (MMP) was analyzed by the JC-1 assay. **(F)** Quantitation of the MMP level indicated by the mean fluorescence intensity (MFI). **(G)** Apoptosis was analyzed by annexin-V PI staining. **(H)** Quantitation of the percentage of apoptotic cells. Data were analyzed by one-way ANOVA, followed by Tukey’s post-hoc test. **P < 0.01, ***P < 0.001, and ****P < 0.0001 compared with the BTZ + shctrl group; ^#^P < 0.05, ^##^P < 0.01, and, ^###^P value < 0.001 compared with the BTZ + shATF3 group.

To confirm the role of ATF3 during BTZ treatment in MM cells, ATF3 expression was modified by shRNA knockdown. ATF3 was successfully knocked down in RPMI-8226 and U-266 cells (**Supplementary Figure 1**). First, shATF3 significantly reduced the increased level of ATF3 mRNA and protein induced by 24 h of BTZ treatment using the IC_50_ dose (**Figures 2A, B**). Considering that the anti-tumor effect of BTZ may be associated with the apoptotic pathway, we measured apoptosis activity after BTZ treatment and the effect of ATF3 on apoptosis. A BTZ-induced increase in the ROS level was attenuated by ATF3 knockdown (**Figures 2C, D**). Similarly, in the JC-1 staining assay, a BTZ-induced elevation in the MMP was reduced by ATF3 knockdown (**Figures 2E, F**). Additionally, BTZ increased the number of apoptotic cells, which was significantly reversed by ATF3 knockdown in the Annexin-PI assay (**Figures 2G, H**).

### BTZ Modulates mRNA Level of ATF3 *via* miR-135a-5p

**Figure 3:**
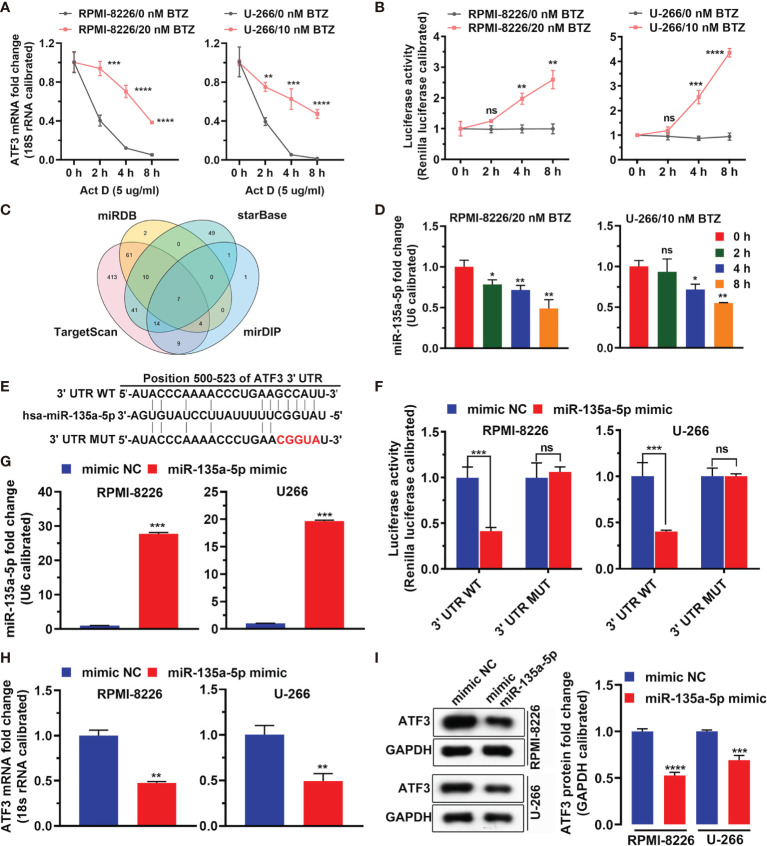
MiRNA regulates ATF3 after BTZ treatment. **(A)** Dynamic level of the ATF3 mRNA change after actinomycin D (Act D) treatment at various doses. Data was analyzed by one-way ANOVA, followed by Tukey’s post-hoc test. **P < 0.01, ***P < 0.001, and ****P < 0.0001. **(B)** Luciferase activity of ATF3 after Act D treatment at various doses. Data was analyzed by one-way ANOVA, followed by Tukey’s post-hoc test. **P < 0.01, ***P < 0.001, and ****P < 0.0001. **(C)** Venn plot of common miRNAs that target ATF3 in TargetScan, miRDB, mirDIP, and DIANA databases. **(D)** Expression of miR-135a-5p was measured by RT-qPCR in cells treated with BTZ. Data were analyzed by one-way ANOVA, followed by Tukey’s post-hoc test. Ns, no significance; *P < 0.05, and **P < 0.01 compared with 0 h **(E)** Conserved and mutant binding sites between the ATF3 3′UTR and miR-135a-5p. **(F)** Dual luciferase reporter assay showing reduced luciferase reporter activity in MM cells that contained the ATF3 3′UTR WT fragment. **(G)** MiR-135a-5p mimics increased miR-135a-5p expression assessed by RT-qPCR. **(H)** The ATF3 mRNA level was reduced by miR-135a-5p mimic transfection as assessed by RT-qPCR. **(I)** Left panel: The ATF3 protein level was reduced by miR-135a-5p mimic transfection in cell lines as measured by western blotting. Right panel: Quantification of ATF3 band intensity, with values normalized to GAPDH. Data are all presented as the mean ± SD. Differences between two groups were analyzed by the Student’s t-test. *P < 0.05, **P < 0.01, ***P < 0.001, ***P < 0.0001. ns, no significance. NC, negative control.

**Figure 3** shows that BTZ reversed Act-D (potent inhibitor of transcription) inhibitory effects on ATF3 mRNA expression (**Figure 3A**). Additionally, the luciferase assay showed that BTZ increased the promoter activity of ATF3 (**Figure 3B**). To determine potential miRNAs that acted as master regulators in this process and miRNAs that targeted ATF3, we screened miRNAs that directly targeted ATF3 mRNA in TargetScan, miRDB, mirDIP, and DIANA databases. As shown in the Venn plot, miR-135a-5p was a common miRNA that targeted ATF3 (**Figure 3C**). Furthermore, the expression level of miR-135a-5p was downregulated in the MM cell lines under BTZ treatment, which was inversely associated with ATF3 mRNA expression (**Figure 3D**). Furthermore, we found conserved binding sites between the ATF3 3′UTR region and miR-135a-5p (**Figure 3E**). Additionally, the dual luciferase reporter assay revealed a significant decrease in luciferase activity in MM cells that were cotransfected with miR-135a-5p and the ATF3-3′UTR luciferase reporter vector compared with the luciferase activity in cells cotransfected with the negative control (mimics ctrl) (**Figure 3F**). The ATF3-3UTR mutant abolished the interaction between miR-135a-5p and the ATF3 3′UTR region in both RPMI-8226 and U-266 cell lines (**Figure 3F**). Furthermore, miR-135a-5p mimics increased miR-135a-5p expression (**Figure 3G**). Accordingly, the levels of ATF3 mRNA and protein were reduced by miR-135a-5p mimic-transfected in cell lines (**Figures 3H, I**). Taken together, the overall action of BTZ in MM cells was associated with ATF3 and miR-135a-5p expression.

### BTZ Modulates Apoptosis Dependently on miR-135a-5p

**Figure 4:**
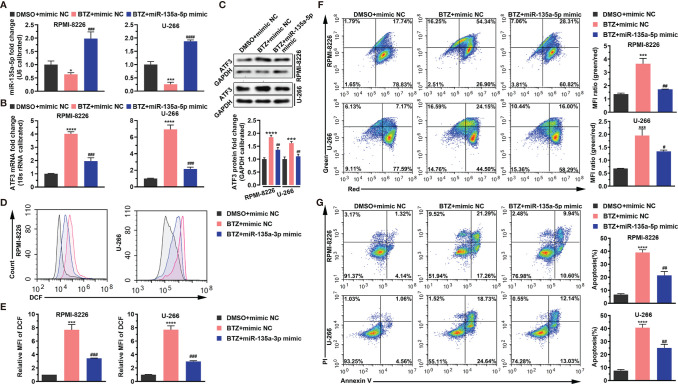
BTZ modulates apoptosis dependently on miR-135a-5p. Apoptosis was analyzed in three groups: **(A)**. DMSO + mimics NC; **(B)** BTZ + mimics NC; **(C)** BTZ + miR-135a-5p mimics. **(A)** The miR-135a-5p level was analyzed by RT-qPCR in RPMI-8226 and U-266 cells of the three groups. **(B)** The ATF3 mRNA level was analyzed by RT-qPCR in RPMI-8226 and U-266 cells of the three groups. **(C)** The ATF3 protein level was analyzed in the three groups. Quantification of ATF3 band intensity, with values normalized to GAPDH. **(D)** The ROS level was measured by the Cellular ROS Assay Kit in the three groups. DCF was detected by FACS. **(E)** Quantitation of the ROS level indicated by DCF. **(F)** Left panel: The MMP was analyzed by the JC-1 assay. Right panel: Quantitation of the MMP level indicated by MFI. **(G)** Left panel: Apoptosis was analyzed by annexin-V PI staining. Right panel: Quantitation of the percentage of apoptotic cells. One-way ANOVA, followed by Tukey’s post-hoc test was used for statistical analysis. ***P < 0.001 and ****P < 0.0001 compared with the BTZ + mimic NC group; ^#^P < 0.05, ^##^P < 0.01, ^###^P < 0.001, and ^####^P < 0.0001 compared with the BTZ + miR-135a-5p mimic group. *P < 0.05 compared with the BTZ+mimic NC group.

Next, we examined the effects of miR-135a-5p on BTZ-induced MM cell apoptosis. The result shows that BTZ reduced the miR-135a-5p level, and this inhibitory effect was rescued by miR-135a-5p overexpression (**Figure 4A**). In addition, we observed that miR-135a-5p mimics resulted in reversing BTZ induced ATF3 mRNA and protein expression levels (**Figures 4B, C**). Furthermore, BTZ-induced increases in ROS levels were reversed by the miR-135a-5p mimics (**Figures 4D, E**). The enhancing effect of BTZ on the MMP was reduced by miR-135a-5p mimics (**Figure 4F**). Moreover, overexpression of miR-135a-5p reversed the BTZ-induced increase in apoptosis (**Figure 4G**).

### BTZ May Regulate CHOP, DR5 and Noxa Expression *via* miR-135a-5p

**Figure 5:**
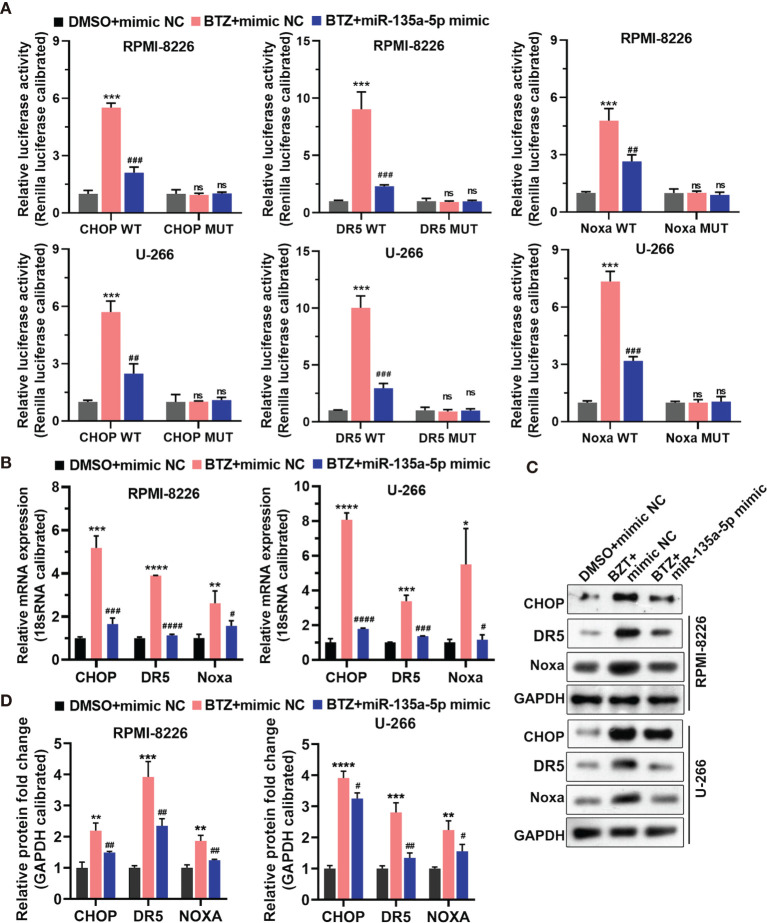
Expression of CHOP, DR5, and Noxa mRNA was analyzed in three groups: **(A)** DMSO + mimics NC; **(B)** BTZ + mimics NC; **(C)** BTZ + miR-135a-5p mimics. **(A)** The relative luciferase activity of CHOP, DR5, and Noxa was detected in RPMI-8226 and U-266 cells of the three groups. **(B)** CHOP, DR5, and Noxa mRNA levels were analyzed by RT-qPCR in the three groups. **(C)** CHOP, DR5 and Noxa protein levels were measured by western blotting in the three groups. **(D)** Quantification of CHOP, DR5, and Noxa band intensity, with values normalized to GAPDH. One-way ANOVA followed by Tukey’s post-hoc test was used for statistical analysis. Ns, no significance; *P < 0.05, **P < 0.01, ***P < 0.001, and ****P < 0.0001 compared with the BTZ + mimic NC group; ^#^P < 0.05, ^##^P < 0.01, ^###^P < 0.001, and ^####^P < 0.0001 compared with BTZ + miR-135a-5p mimic group.

To explore the signaling pathways through which BTZ modulated MM cell apoptosis, we examined the CHOP transcription factor related to endoplasmic reticulum (ER) stress-induced apoptosis, CHOP-dependent death receptor 5 (DR5), and Noxa, a proapoptotic BH3-only protein. The results showed that miR-135a-5p reversed the increases in promoter activities of CHOP, DR5, and Noxa mediated by BTZ and their mutants decreased the activities (**Figure 5A**) Furthermore, miR-135a-5p attenuated the BTZ-induced increases in CHOP, DR5 and Noxa mRNA levels (**Figure 5B**). Similarly, miR-135a-5p reversed the enhancing effects of BTZ on CHOP, DR5 and Noxa protein levels (**Figures 5C, D**).

## Discussion

MM is a life-threatening cancer that can be effectively treated by BTZ. However, BTZ resistance may develop over the course of therapy. Full exploration of the detailed mechanism of BTZ effectiveness and tolerance in MM would be beneficial to patients. In the present study, we found that BTZ inhibited cell proliferation and expression of ATF3 was significantly increased under BTZ treatment in a dose-dependent manner. This indicated that ATF3 might function as an important regulator in BTZ effectiveness against MM. This result was consistent with a previous report in which BTZ resistance was increased after silencing ATF3 expression (25).Additionally, expression of ATF3 is closely related to the BTZ treatment response and progression-free survival (25). We also found that knockdown of ATF3 reduced the effects of BTZ on apoptosis, which further supports the hypothesis that ATF3 plays an important role in BTZ treatment of MM.

It was very interesting to observe the co-occurrence of increased apoptotic and necrotic cells induced by BTZ, which was partially blocked by shATF3. We believe that BTZ-induced apoptosis can be considered a hallmark of MM treatment. In the BTZ treatment process, MM cells defensively trigger non-apoptotic cell death pathways, such as necrosis, autophagy, and mitotic catastrophe, to resistant apoptosis induced by BTZ. Such tumorigenesis may be associated with mitotic kinases, the ATP level, and mitochondria. While co-occurrence of apoptosis and necrosis is poorly understood, we consider that such a condition is mediated by the network of internal/external stress factors and ATP/plasma membrane permeabilization and the MMP. In the current study, it was surprising that ATF3 knockdown caused a significant decrease in necrosis induced by BTZ in U266 cells, but not RPMI-8226 cells, which suggested a distinct role of ATF3 in the different MM cell lines. Therefore, we should further investigate the role of ATF3 in death signal stimulation to reveal shared pathways.

Furthermore, we identified miR-135a-5p as an miRNA that directly targeted ATF3 mRNA and downregulated its expression under BTZ treatment, which indicated that miR-135a-5p may act as a regulatory microRNA. Finally, we investigated the effects of miR-135a-5p and ATF3 mRNA on BTZ treatment of MM cells. The results demonstrated that miR-135a-5p reduced the effects of BTZ on the apoptotic process, which might influence expression of CHOP, DR5, and Noxa proteins. It is well recognized that transcriptional activation of CHOP is a major event in ER stress-mediated apoptosis. Moreover, ER stress-induced CHOP activation is associated with DR5 in human cancer cells. CHOP-mediated DR5 transcription is dependent on several transcription factors such as ATF3 and ATF4, Therefore, we determined whether BTZ-induced apoptosis was mediated through the ATF3-CHOP/DR5 axis. Additionally, Noxa gene expression is mediated by ATF3 in Noxa-dependent intrinsic apoptosis, i.e., the ATF3-CHOP/Noxa axis. For example, the CHOP/NOXA/Mcl-1 axis contributes to LiCl-induced apoptosis (26), and ATF4-triggered Noxa- and DR5-dependent extrinsic apoptosis have been seen in fangchinoline-treated esophageal squamous cells (27). Because ER stress in BTZ-treated MM cell lines is beyond their self-defense capacity, the MM cell activates apoptotic signals to remove damage organelles (28). We hypothesize that CHOP/Noxa and CHOP/DR5 axes may be triggered by an ATF3-dependent pattern.

ATF3 is highly prominent in cancer that includes breast, laryngeal, and lung cancers (29). Conversely, other studies have demonstrated that ATF3 functions as a tumor suppressor in other cancers that include esophageal, colon, and bladder cancers (30). In addition, ATF3 and Smad are synergistically involved in JNK-induced apoptosis of MM cells (31). In the brain, BTZ induces oxidative stress, mitochondrial dysfunction, and apoptosis by the activation of ATF3 (32). Moreover, and enhanced interaction between c-Jun and ATF3 promotes BTZ-induced mechanical allodynia (4).

An miRNA can regulate numerous genes and act as either a tumor suppressor or oncogenic miRNA. In MM, combined overexpression of miR-520g and miR-520h inhibits bortezomib-resistant tumor growth *in vivo (* 19). Expression of miR-135a-5p has been found in patients with colorectal cancer (33) and acts as a mechanistic regulator in patients with HCV (34). Additionally, miR-135a-5p inhibits the proliferation of head and neck cancer cells and promotes apoptosis (35). Moreover, miR-135a-3p inhibits ovarian tumorigenesis by regulating CCR2 (36). The proliferation, migration, and invasion of glioma cells are regulated by the miR-135a-5p axis, which provides viable therapeutic avenues for the treatment of glioma (37). MiR-135a-5p may also act as a tumor suppressor in the development of colorectal adenomas and promote apoptosis of colon cancer cells (38). Here, we demonstrated that miR-135a-5p also targeted ATF3 (39). Our results also revealed the importance of miR-135a-5p in BTZ drug sensitivity in MM.

The present study has some limitations such as a lack of rescue experiments and interference experiments of ATF3 mRNA and protein in the cell lines. The MM cell lines for functional assessment were also limited and there was a shortcoming of evidence from BTZ-resistant MM cells in terms of the regulation of ATF3 and miR-135a-5p. Overall, our results suggested crosstalk between miR-135a-5p and ATF3 in BTZ-induced apoptosis and necrotic cell death. It is unknown whether the regulation was in a dual direction or only one direction. Although the detailed association of BTZ and crosstalk of miR-135a-5p and ATF3 remain to be explored further, to the best of our knowledge, this study is the first to demonstrate an interaction of ATF3 and miR-135a-5p in BTZ treatment of MM. In conclusion, these results revealed miR-135a-5p/ATF3 crosstalk in BTZ treatment of MM and suggests a new regulatory protein interactome to potentially provide a therapeutic option for MM.

## Data Availability Statement

The raw data supporting the conclusions of this article will be made available by the authors, without undue reservation.

## Author Contributions

XL, Rc, and ZC made substantial contributions to the concept and design of the study as well as the manuscript. XL, CH, XN, QC, YT, XF, YL, CN, XX, and XW conducted the experiments and data analysis. All authors contributed to the article and approved the submitted version.

## Funding

The present study was supported by grants from the Natural Science Foundation of Fujian Province (grant no. 2018J05151 and 2019J01626).

## Conflict of Interest

The authors declare that the research was conducted in the absence of any commercial or financial relationships that could be construed as a potential conflict of interest.

## Publisher’s Note

All claims expressed in this article are solely those of the authors and do not necessarily represent those of their affiliated organizations, or those of the publisher, the editors and the reviewers. Any product that may be evaluated in this article, or claim that may be made by its manufacturer, is not guaranteed or endorsed by the publisher.
