# Supplementary material for: Bortezomib Inhibits Multiple Myeloma Cells by Transactivating ATF3 to Trigger miR-135a-5p- Dependent Apoptosis
Source: Front Oncol. 2021 Sep 22;11:720261. doi: 10.3389/fonc.2021.720261 (PMC8493032; doi:10.3389/fonc.2021.720261)
Supplement: Supplementary file 1 [file DataSheet_1.docx]

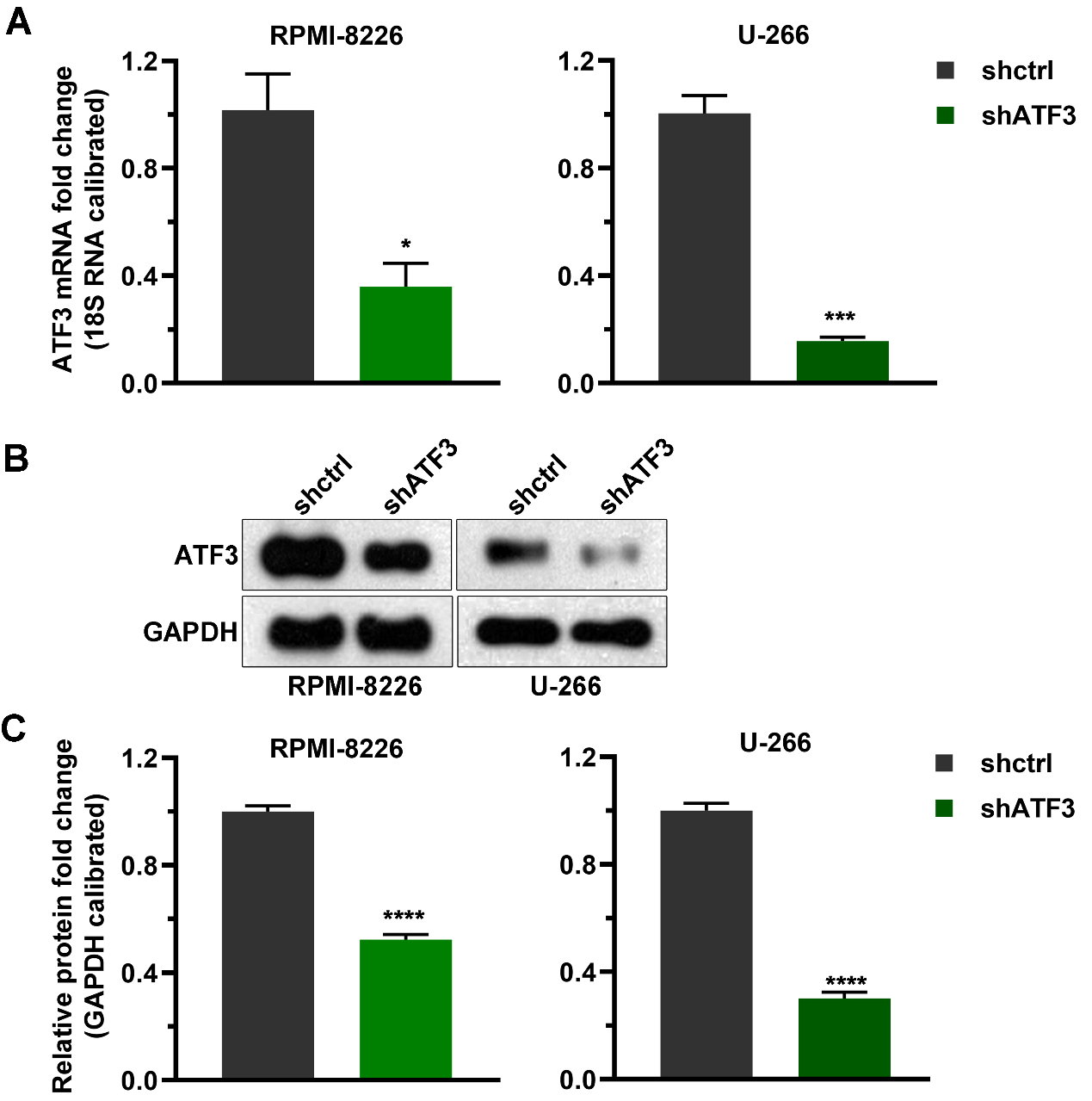


Supplementary figure 1. ATF3 was successfully knocked down in RPMI-8226 and U-266 cells. (A) The knockdown efficiency of shATF3 was evaluated using RT-qPCR. (B) ATF3 protein level was detected using western blotting. GAPDH was used as a loading control. (C) Quantification of ATF3 band intensity with values normalized to GAPDH. Unpaired Student’s t-test. *P < 0.05, ***P < 0.001****P < 0.0001.
