# Supplementary material for: Bortezomib Inhibits Multiple Myeloma Cells by Transactivating ATF3 to Trigger miR-135a-5p- Dependent Apoptosis
Source: Front Oncol. 2021 Sep 22;11:720261. doi: 10.3389/fonc.2021.720261 (PMC8493032; doi:10.3389/fonc.2021.720261)
Supplement: Supplementary file 2 [file Table_1.docx]

Supplemental table 1. Primer sequence for RT-qPCR.

| Gene name | Sequence (5’-3’) |
| --- | --- |
| U6-RT | AACGCTTCACGAATTTGCGT |
| U6-F | CTCGCTTCGGCAGCACA |
| U6-R | AACGCTTCACGAATTTGCGT |
| has-miR-135a-5p-RT | GTCGTATCCAGTGCAGGGTCCGAGGT  ATTCGCACTGGATACGACTCACAT |
| has-miR-135a-5p-F | CGCGTATGGCTTTTTATTCCT |
| has-miR-135a-5p-R | AGTGCAGGGTCCGAGGTATT |
| 18s-F | AGGCGCGCAAATTACCCAATCC |
| 18s-R | GCCCTCCAATTGTTCCTCGTTAAG |
| ATF3-F | CGCTGGAATCAGTCACTGTCAG |
| ATF3-R | CTTGTTTCGGCACTTTGCAGCTG |
| CHOP-F | GGTATGAGGACCTGCAAGAGGT |
| CHOP-R | CTTGTGACCTCTGCTGGTTCTG |
| DR5-F | AGCACTCACTGGAATGACCTCC |
| DR5-R | GTGCCTTCTTCGCACTGACACA |
| Noxa-F | CTCGATGCAGAGACAGAGGTCG |
| Noxa-R | AGGAGCCTGTTTGCCAACTTGC |

RT: Primer for reverse transcription; F: Forward primer; R: Reverse primer.

Supplemental table 2. Antibody information

| Antibody name | Host | Supplier | Cat. No |
| --- | --- | --- | --- |
| ATF3 | Rabbit | Abcam | ab176872 |
| CHOP | Rabbit | Proteintech | 15204-1-AP |
| DR5 | Rabbit | Proteintech | 15497-1-AP |
| Noxa | Mouse | Abcam | ab68523 |
| GAPDH | Rabbit | CST | #2118 |
| HRP Anti-Rabbit IgG(H+L) | Goat | Proteintech | SA00001-2 |
| HRP Anti-Mouse IgG(H+L) | Goat | Proteintech | SA00001-1 |

Supplemental table 3. Primers for gene cloning

| Gene name | Sequence (5’-3’) |
| --- | --- |
| ATF3 shRNA-F | CCGGGTTGTGCTTTCTAGCAAATATCTCGAGATATTTGCTAGAAAGCACAACTTTTT |
| ATF3 shRNA-R | AATTAAAAAGTTGTGCTTTCTAGCAAATATCTCGAGATATTTGCTAGAAAGCACAAC |
| PmirGLO-ATF3-F | AGCTCGCTAGCCTCGAGCAGTCGTGGTATGGGGGCG |
| PmirGLO-ATF3-R | CATGCCTGCAGGTCGACTTTACCATGTCTTGGTTC |
| PmirGLO-ATF3-MUT-F | ACCCTGAACGGTATGGAGAGCTGTCTTCCTG |
| PmirGLO-ATF3-MUT-R | CTCTCCATACCGTTCAGGGTTTTGGGTATAAA |
| PmirGLO-CHOP-F | CTAACTGGCCGGTACCTTGTTTGTTTGTTTTGTTTTTTG |
| PmirGLO-CHOP-R | TCTTGATATCCTCGAGGCCTTAGACTTAAGTCTCTG |
| PmirGLO-CHOP-MUT-F | TGCAGACACCGGTTGCCAAACCCGCCCCCCTTTCC |
| PmirGLO-CHOP-MUT-R | TTTGGCAACCGGTGTCTGCA |
| PmirGLO-DR5-F | TAACTGGCCGGTACCACACGCCGCCTTTAAGAACTG |
| PmirGLO-DR5-R | TCTTGATATCCTCGAGGACTTGGGGCGGCGCGGCTG |
| PmirGLO-DR5-MUT-F | GGAATGAAAATACATAACAATGTTTTATTTCCTAGACTAG |
| PmirGLO-DR5-MUT-R | TTGTTATGTATTTTCATTCC |
| PmirGLO-Noxa-F | TAACTGGCCGGTACCGGGAAGGCTACAGTGGGTAAG |
| PmirGLO-Noxa-R | TCTTGATATCCTCGAGGGTGACGTAGGGAAACTAGAC |
| PmirGLO-Noxa-MUT-R | TCTTGATATCCTCGAGAAACTAGACGCCCGGCCCGCCCC |

F: Forward primer; R: Reverse primer.
